# Managing Ureteropelvic Junction Obstruction in the Young Infant

**DOI:** 10.3389/fped.2020.00242

**Published:** 2020-05-27

**Authors:** Niccolo Maria Passoni, Craig Andrew Peters

**Affiliations:** University of Texas Southwestern Medical Center, Dallas, TX, United States

**Keywords:** robotic assisted pyeloplasty, infant–age, prenatal hydronephrosis, diuretic nephrogram, ureteropelvic junction (UPJ) obstruction

## Abstract

In the last decade, management of congenital UPJ obstruction has become progressively observational despite the lack of precise predictors of outcome. While it is clear that many children will have resolution of their hydronephrosis and healthy kidneys, it is equally clear that there are those in whom renal functional development is at risk. Surgical intervention for the young infant, under 6 months, has become relatively infrequent, yet can be necessary and poses unique challenges. This review will address the clinical evaluation of UPJO in the very young infant and approaches to determining in whom surgical intervention may be preferable, as well as surgical considerations for the small infant. There are some clinical scenarios where the need for intervention is readily apparent, such as the solitary kidney or in child with infection. In others, a careful evaluation and discussion with the family must be undertaken to identify the most appropriate course of care. Further, while minimally invasive pyeloplasty has become commonly performed, it is often withheld from those under 6 months. This review will discuss the key elements of that practice and offer a perspective of where minimally invasive pyeloplasty is of value in the small infant. The modern pediatric urologist must be aware of the various possible clinical situations that may be present with UPJO and feel comfortable in their decision-making and surgical care. Simply delaying an intervention until a child is bigger may not always be the best approach.

## Introduction

Hydronephrosis is the most commonly diagnosed genito-urinary abnormality on prenatal ultrasounds (1). In the past, corrective surgery was offered to every child who presented with uretero-pelvic junction obstruction (UPJO). Indeed, prior to diffusion of fetal ultrasound, patients with this condition were identified due to their signs and symptoms (2). Children were commonly diagnosed between the ages of 6 and 15 years, with only 14% of them being younger than 1 year of age (3).

However, prenatal imaging has increased the rate of diagnosis of asymptomatic cases that may not have otherwise not been detected until later in life. A multitude of studies has questioned the earlier operative paradigm by demonstrating a high rate of spontaneous resolution. Unfortunately, this culminated in a conundrum which today still has no clear solution: which asymptomatic infant with hydronephrosis will lose precious renal function if left untreated?

Finally, diagnosis of a UPJO that warrants surgical correction in an infant poses technical challenges in the modern era of minimally invasive surgery.

In this chapter the threats posed by chronic obstruction to the kidney as well as the natural history of prenatally diagnosed UPJO will be discussed. Surveillance algorithms aimed at identifying early candidates for surgery will be described prior to introducing novel markers and imaging methodologies to improve risk stratification. In the end, traditional and minimally invasive approaches will be compared with a particular attention to tips and tricks for the infant patient.

## The Effects of Obstruction on the Kidney

Hydronephrosis is an abnormal dilation of the collecting system. However, not all hydronephrosis is associated with clinically significant obstruction that will lead to renal function deterioration (4). Unfortunately, long-term complications of renal damage may not be evident until the patient reaches adulthood. Even if a child has normal renal function, these patients are four times more likely to develop ESRD (5) and can require renal replacement therapy in young adulthood (6). Nephrogenesis terminates at 36 weeks of gestation, without any more nephrons formed after birth. Premature babies will have a lower number of nephrons compared to children born at term (7). Therefore, any insult that leads to renal injury will not be followed by replacement of damaged nephrons but by adaptive changes of the remaining nephrons (8). While this mechanism maintains glomerular filtration rate at first, in the long term it appears to lead to renal damage in both the obstructed and the contralateral kidney, as shown in murine models (9).

Furthermore, obstruction that originates *in utero* can lead to more deleterious effects by altering the pathways of renal development (10, 11).

Initial series of biopsies obtained at time of surgical repair showed that up to 21% of children with a differential renal uptake (DRU) on diuretic renography >40% at time of surgery had histological changes, while only 34% of patients with a DRU <40% had normal findings (12). Interestingly, when grouping children by presentation, they demonstrated that only 19% of children diagnosed due to symptoms harbored moderate or severe histological changes, compared to 50% of children diagnosed either prenatally or due to a palpable mass (up to 80%). A larger more recent study found that 67% of biopsies from 61 children had glomerular sclerosis (13). Interestingly, the number of affected glomeruli did not significantly correlate with either degree of hydronephrosis nor DRU. In addition, tubulointerstitial changes were found in only 26% of patients, and significant fibrosis was more common in patients older than 1 year of age, suggesting a potentially progressive process with chronic obstruction. Alteration of the renal parenchyma secondary to obstruction has been documented in human fetuses as well. An autopsy study conducted on fetuses with evidence of UPJO on prenatal ultrasound showed that obstructed kidneys have a reduction in glomerular number and cortical thickness as well as an increase in fibrosis when compared to specimens from age-matched fetuses with normal kidneys (14). The authors found that fibrosis and reduced glomerular numbers correlated strongly with hyperechogenicity on prenatal ultrasound, which is consistent with clinical observations.

In reality, damage from obstruction is likely secondary to partial obstruction that develops later in pregnancy once nephrogenesis is almost complete, otherwise it would lead to cystic dysplasia or renal agenesis (15).

Just as not all hydronephrosis will persist, as will be discussed in the next section, not all obstructed systems harbor the same damage potential. Therefore, the clinician must be able to synthesize the clinical history and diagnostic data to identify the child more at risk of losing renal function.

## Natural History of Prenatally Detected Hydronephrosis

While at first it was believed that most UPJ obstructions with severe dilation detected prenatally required intervention, several studies have shown a relatively high rate of spontaneous resolution. This has led to a shift in management, centered on the serial monitoring of renal dilation and function to hopefully identify the children that will eventually require surgery as early as possible without irreversible loss of renal functional potential.

Several statistics are useful when counseling families of newborns with hydronephrosis secondary to UPJO.

First, that rates of resolution and/or improvement even for severe dilation, as in grade 3 and 4 as defined by the Society for Fetal Urology (SFU) are reasonably good. Indeed, complete resolution rates in observed children range from 33 to 70% (16–23). In the literature, lower rates of resolution are associated with more severe hydronephrosis. Furthermore, another important parameter is improvement in hydronephrosis. Indeed, a change in SFU Grade from 3–4 to 1–2 is considered significant and likely reflects a kidney without significant risk of functional deterioration.

Second, not all children with moderate and severe hydronephrosis have poor DRU as measured on diuretic renography. Data from studies shows that between 10 and 39% of children with SFU grade 3 or 4 have a reduced DRU at diagnosis, defined as <40%. These children are usually offered early pyeloplasty. However, if observation is performed for kidneys with a DRU <40%, renal function remains stable in ~80% of them at 1 year (17, 24). It remains undefined how many may experience later deterioration without intervention.

## Diagnosis and Initial Evaluation

The challenges in managing infants with prenatally detected UPJO are secondary to a lack of diagnostic tools that can identify obstruction that will lead to deterioration of renal function or prevent normal renal functional development. To further complicate matters, the current gold standard evaluation of renal function is diuretic renography; however, we are not sure if a kidney with “normal” DRU on nephrography can be considered completely normal since it likely has received some insults from *in-utero* obstruction (13). Also, diuretic renography does not provide any information regarding the multiple other important functions of the kidney, including tubular homeostatic and endocrine functions. Therefore, urologists need to rely on a combination of ultrasound and diuretic renography findings to individualize management.

**Figure 1:**
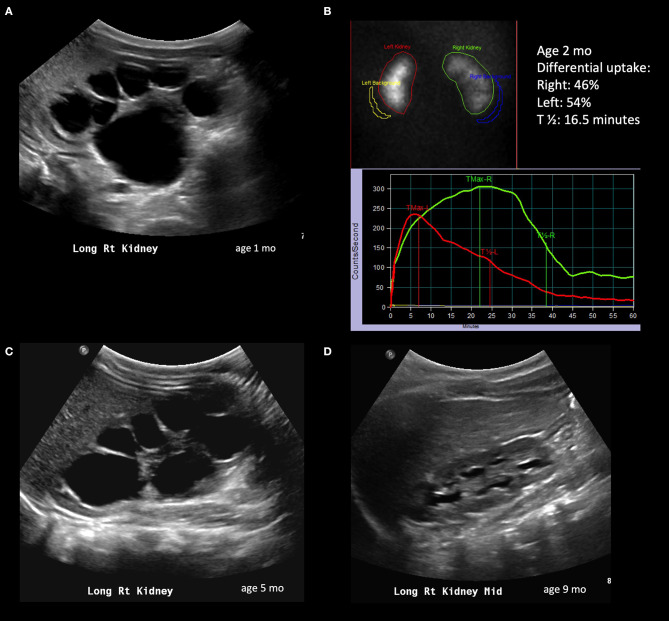
Radiological history of an infant with resolution of severe hydronephrosis. **(A)** Postnatal ultrasound confirming prenatally diagnosed hydronephrosis; the image shows SFU grade 4 hydronephrosis with thinned isoechoic parenchyma. **(B)** Diuretic renogram performed at 2 months of age, showing symmetric uptake; despite the right kidney exhibits a delayed washout curve, it is still considered adequate. **(C)** Repeat ultrasound at 5 months showing persistent SFU grade 4 hydronephrosis. **(D)** Follow-up ultrasound at 9 months showing significant spontaneous improvement of hydronephrosis; the child will still however need follow-up imaging to ensure persistent improvement.

Once a baby with prenatal hydronephrosis is delivered, a postnatal ultrasound is obtained to assess persistence of hydronephrosis. Usually it is obtained between 48 and 72 h after birth, due to transient neonatal dehydration. However, it is recommended to obtain this study earlier in specific cases, such as bilateral hydronephrosis, solitary kidney or a history of oligohydramnios. It is also important to record size of the kidney, thickness and echogenicity of the renal parenchyma, as well as appearance of the bladder and post void residuals. Important information can be obtained from the initial ultrasound. Severe hydronephrosis is associated with diffuse and uniform dilation of the calyces with flattening of the renal papillae (Figure 1). In severe cases, the hydronephrosis leads to thinning of the renal parenchyma. Asymmetric dilation should raise suspicion of a duplicated system. It is important to remember that a severely dilated renal pelvis with mild-to-moderate dilation of the intra-renal collecting system is usually a hallmark of milder obstruction and pelvic dilation should not be used to draw therapeutic conclusions by itself. Isolated dilation of the extra-renal pelvis is usually considered a benign finding (25). One study showed that while infants extra-renal pelvis dilation have slightly higher rates of UTI, it resolves in 98% of patients on follow-up (26). Furthermore, dilation of the intra-renal pelvis has by far as more significant prognostic values, as highlighted by the adoption of anterio-posterior renal pelvis diameter by the UTD classification (27). Finally, the presence of a dilated ureter can indicate either the presence of vesicoureteral reflux or a more distal obstruction.

A voiding cystourethrogram is usually obtained to rule out lower urinary tract obstruction or vesicoureteral reflux, especially for cases with bilateral hydronephrosis, unilateral hydronephrosis in solitary kidneys or oligohydramnios. However, for a child with unilateral renal dilation without ipsilateral ureteral involvement, a voiding cystourethrogram to rule out reflux might not be needed. Indeed, a recent review showed that among children with UPJO the pooled prevalence of vesicoureteral reflux is 8.2%, 3-fold higher than in children without UPJO (28). Lee et al. demonstrated a higher rate of vesicoureteral reflux among patients with higher grades of hydronephrosis. With the goal or reducing unnecessary radiation exposure and testing, they developed a risk-based approach based on ultrasound findings such as presence of duplication, hydroureteronephrosis and renal dysplasia (29). Performing a VCUG only in children with all three aforementioned ultrasound criteria would reduce the number of tests ordered by 40% while maintaining the same miss rate of reflux as if ordering a VCUG only if severe hydronephrosis were present. However, it remains controversial as to how valuable the VCUG and identification of reflux might be in this population.

## Diuretic Nephrography

Once severe hydronephrosis is confirmed, diuretic renography is ordered to assess the degree of obstruction as well as the level of renal function. Technetium-99m (99mTc) mercaptoacetyltriglycine (MAG3) is the preferred radionuclide due to its short half-life and its excretion via both glomerular filtration and active tubular secretion, allowing for assessment of poorly functioning kidneys. In general, the amount of tracer uptake in the first 2 min after injection correlates with the glomerular filtration rate.

Several elements can be controlled in order to obtained the most informative study (3). The patient should be adequately hydrated prior to the procedure. Second, the bladder should be emptied with a catheter since a full bladder can impair upper tract drainage, as well-increasing gonadal radiation exposure. Finally, the diuretic has been described as being administered 15 min prior to radionuclide injection, at the same time, or 20–30 min after. The most common approach includes administration of the diuretic once the entire dilated collected system is filled with radionuclide in order to better assess washout. The diuretic that is commonly used is furosemide, at a dose of 1 mg/kg for infants.

Diuretic renography provides several useful parameters. First, it estimates differential renal function. It has been shown that unilateral variation within 5% is considered physiological (30), while a loss >5% should be considered as loss of renal function (31). Second, washout curves can be interpreted to assess the degree of obstruction. However, the traditional T1/2 cut-offs used for obstruction should not be used as rigid checkpoints for therapeutic algorithms.

**Figure 2:**
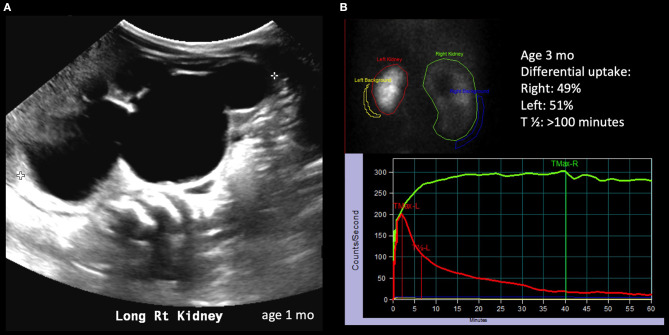
Radiological history of an infant with severe hydronephrosis secondary to significant UPJ obstruction. **(A)** Ultrasound showing SFU grade 4 hydronephrosis on postnatal imaging. **(B)** Diuretic renogram showing symmetrical uptake; unlike the case described in Figure 1, the drainage curve of the right kidney does not show any drainage, even after diuretic administration.

**Figure 3:**
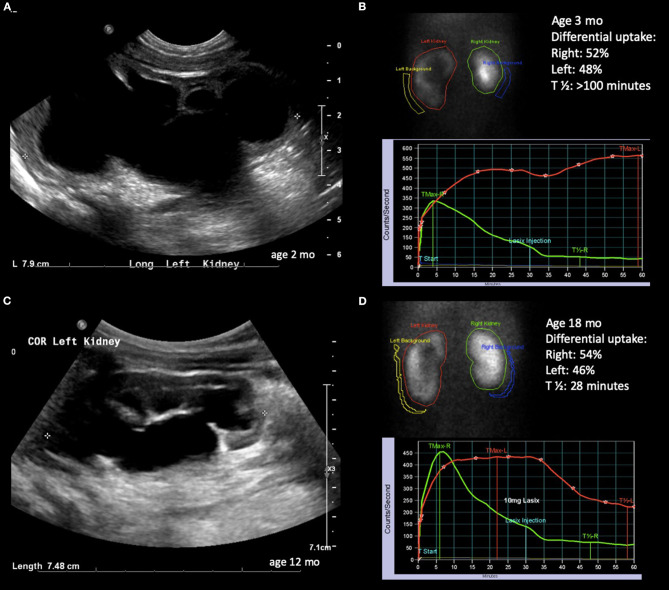
Radiological history of an infant with severe hydronephrosis secondary to UPJ obstruction and biphasic diuretic curve. **(A)** Ultrasound confirming SFU grade 4 hydronephrosis at 2 months of age. **(B)** Diuretic renogram showing symmetrical uptake with biphasic washout curve. **(C)** Ultrasound at 12 months of age showing slightly improving but still SFU grade 4 hydronephrosis. **(D)** Improved washout curve on diuretic renogram, but still with delayed emptying.

The astute clinician should remember that UPJO can be a dynamic process and should consider changes in degree of hydronephrosis, DRU as well as washout curves when formulating a treatment strategy. The washout curve can show prompt drainage (Figure 1) or obstruction if a flat, plateauing shape is seen (Figure 2). However, a biphasic curve can show dynamic obstruction. This curve normally shows prompt drainage that eventually plateaus or even raises, suggesting varying degrees of obstruction (Figure 3). Finally, delayed cortical transit time, which is defined as the absence of radionuclide in the sub-cortical renal parenchyma within 3–8 min of injection has been associated with outcomes after pyeloplasty. Song et al. demonstrated that children with delayed cortical transit time had greater improvement of their DRU after surgery compared to those with normal values (32). Furthermore, a delayed transit time has been shown to be a prognostic factor identifying which children will progress to surgery while on observation (33, 34). Interestingly, in a porcine model, delayed cortical transit time has been linked to histological changes such as glomerulosclerosis, decreased number of glomeruli, tubular atrophy, and increased fibrosis (35). However, the correlation between hydronephrosis and diuretic renography findings is poor (36). Among 13% of children with improving or stable hydronephrosis, DRU worsened more than 5%.

## Biomarkers

For a long time there has been a focus on urine biomarkers to screen for children with UPJO who will ultimately develop renal damage, yet none are in regular clinical practice to date. Kostic et al. sampled urine and blood from newborns with either lower or upper urinary obstruction and compared values of biomarkers with healthy infants matched by gender and gestational age (37). They identified NGAL (Neutrophil Gelatinase-Associated Lipocalin), RBP (Retinol Binding Protein), TGF-ß1 (Transcription Growth Factor-ß1), and KIM-1 (Kidney Injury Molecule-1) as promising markers, compared to serum creatinine and cystatin, for identifying which patients with unilateral hydronephrosis will progress and require surgery. All their values decreased after surgery.

These proteins are markers of ischemic and tubule-interstitial pathology, and herald renal damage prior to radiological findings.

The benefits of using urine is that it is readily available, can be collected longitudinally and in a non-invasive manner. However, voided urine contains a mix of urine from both kidneys, and markers from an obstructed system can easily be diluted. Froelich et al. performed urine proteomics analysis by sampling urine from both the obstructed kidney at time of surgery and the bladder (38). They identified 76 proteins that were present both in renal and bladder samples, showing that obstruction produces changes in the urine proteome that are also secondary to compensatory changes in the non-obstructed kidney. A significant number of these proteins were part of the oxidative stress pathway, underling its important role in the pathogenesis of UPJO. Future areas of development for novel biomarkers are magnetic resonance imaging and proteomics and metabolomics (39). While the latter can provide quantitative information on glomerular numbers and volume, the former still requires generation of age-specific normative-data.

Further multi-disciplinary and multi-institutional studies are needed however to identify a marker that can reliably identify obstructed renal units that are at risk of deterioration.

## Clinical Risk Factors of Renal Deterioration

A copious literature exists investigating what factors are able to predict renal deterioration and thus identify candidate who would benefit from early surgery. This is based on the belief that operating on a child whose DRU has not deteriorated yet will lead to better long-term results. However, there are reports showing that lost function during observation will be recovered after surgical correction (18) and will last into puberty (40). Furthermore, early detection of surgical candidates can potentially reduce costs of follow-up imaging as well as stress for the families. The prediction usually relies on parameters obtained from ultrasound and nuclear medicine imaging. With regard to renal function, infants with a >10% difference in DRU between then hydronephrotic kidney and contralateral healthy one at initial evaluation has been found to experience renal deterioration 3 times more often and to be two times more likely to develop symptoms (41). As mentioned earlier, delayed cortical transit time has been found to be a predictor of deterioration, once having adjusted for other factors such as DRU, T1/2 and hydronephrosis (32, 42). Anterior-posterior diameter (APD) on initial ultrasound has been found to be an independent predictor of resolution of hydronephrosis (43). An APD of 24 mm an initial evaluation has been shown to have high specificity and sensitivity to predict need of surgery, secondary to either a drop of 10% or greater in DRU or worsening hydronephrosis with an obstructed nephrogram (44).

The idea of creating a variable that includes information from both degree of hydronephrosis severity and renal parenchyma thinning was first introduced by Shapiro and coworkers with hydronephrosis index (45). This was obtained by subtracting the area of the calyces and renal pelvis from the total area of the kidney and then dividing it by the total area. The hydronephrosis index was shown to be easily reproducible and associated with resolution or worsening of hydronephrosis. Later, Cerrolaza et al. (46), by applying machine learning to ultrasound images and diuretic renogram curves, described how quantitative analysis of renal ultrasound images can predict diuretic renography curves and help reduce the number of nuclear medicine studies. More recently, Rickard et al. (47) showed that the renal parenchyma to hydronephrosis area ratio correlates well with DRU and T1/2, and demonstrated a very good performance in selecting children who will require surgery.

## Treatment Algorithm

**Figure 4:**
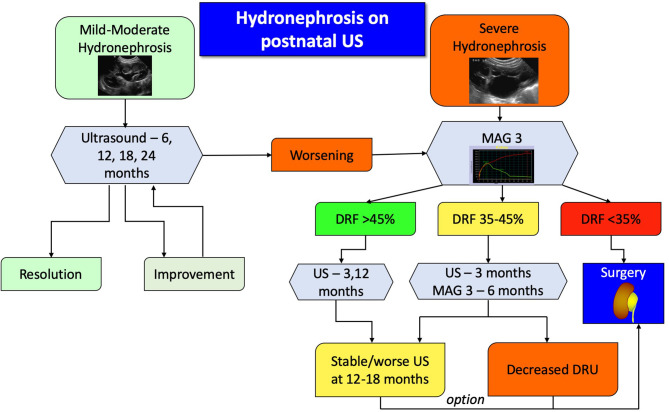
Proposed algorithm for therapeutic approach to post-natal asymptomatic hydronephrosis. Antibiotic prophylaxis is recommended for severe hydronephrosis during the first year of life.

Absolute candidates for surgical treatment are symptomatic patients, however defining symptoms of UPJO in infants can be challenging, since most won't be able to complain about pain. Significant symptoms also include recurrent urinary tract infections despite antibiotic prophylaxis, hematuria, kidney stones, or mass effect from the severely dilated kidney. Another absolute indication for surgery is the child with clinical obstruction in a solitary kidney and evidence of reduced overall renal function. For all other patients, the algorithm (Figure 4) is a suggested clinically pragmatic approach.

In essence, patients with mild or moderate hydronephrosis, defined as SFU grade 1 or 2, should be followed with serial ultrasounds to detect either improvement and resolution or progression. Children with confirmed severe hydronephrosis on post-natal imaging or those who progress to it should undergo a MAG3 study. The findings of the diuretic renography will dictate the follow up. If the DRU is more than 45%, ultrasound is repeated to assess for degree of dilation, whereas if the DRU is <35% surgery is usually recommended. If the hydronephrosis does not improve on repeated ultrasound images, despite a normal DRU, then surgery can be appropriately offered. For all other patients, US and MAG3 should be repeated, and in case of worsening hydronephrosis or DRU the surgery should be considered. It has been shown that if a patient demonstrates two consecutive drops in their DRU on two consecutive scans, then there is an 85% he or she will require surgery (48). As mentioned earlier, a delayed cortical transit time is also a strong indicator of future renal deterioration and should be incorporated into clinical algorithms without having to wait for actual loss of function.

If the DRU is <10%, some authors argue that a nephrectomy is indicated where there is development of symptoms such as infections or hypertension; otherwise non-intervention is warranted. There are reports of a significant improvement of function in kidneys with a pre-operative DRU <10%. Wagner et al. reported a return to function to a range of 27–53% at 1 year after pyeloplasty (49). Other authors instead recommend placement of a nephrostomy tube for 4 weeks to determine if there will be some recovery of renal function. In their series, up to 70% of kids treated with a urinary diversion recovered from <10% DRU to an average or 29% (50, 51). While this can occur, significant functional improvement has not been common in the senior author's experience, raising concern as to the validity of initial functional assessments, which can be problematic.

## Surgical Treatment of UPJO in the Infant Patient

The challenges of surgical correction of UPJO in patients younger than 1 year of age mainly reside in the adaptation of minimally invasive techniques due to the patient's size. The open Anderson-Hynes dismembered pyeloplasty is considered the gold standard approach in this population. In the very small infant, the dorsal lumbotomy is preferred by the authors for several reasons. First, it avoids muscle splitting, reducing post-operative pain. Second, it allows for direct access to the posterior aspect of the renal pelvis and ureter. Finally, the incision is in a more discrete area, compared to a lateral approach. However, the open approach, unlike the laparoscopic or robotic ones, does not allow for access to the entire ureter, in case of a longer than expected stricture. Another potential disadvantage of the open approach is the use of excessive traction on the tissues in order to improve exposure toward the surgical incision. Indeed, the minimally invasive approach allows to bring the instruments to the tissues, instead of having to bring the tissues to the surgical site, avoiding unnecessary tension that could damage the ureter. Furthermore, unless for selective circumstances like a posterior renal pelvis, minimally-invasive pyeloplasty allows for surgical correction without the need to rotate the kidney.

Minimally invasive approaches have shown direct patient benefit in terms of reduced hospital stay, reduced need of pain medications and improved cosmetics results in older children (52). However, both laparoscopic and robotic pyeloplasties were originally received with skepticism in infant populations. The main concerns raised by critics were the smaller operative field offered by an infant pneumoperitoneum, the limited space for port placement, lack of appropriate-sized instruments for the robot and finally the fact that the robotic system would limit access to the patient by the anesthesia team. It was also argued, appropriately to some degree, that there was limited benefit for the costs in time and instrumentation.

The first ever description of a successful laparoscopic dismembered pyeloplasty in the pediatric literature was by the senior author in 1995, on a 7 years old child (53). Subsequently, Dr. Tan (54) reported on a series of 16 children, in which two had persistent post-operative obstruction, and both these patients were 3 months of age at time of surgery. Hence laparoscopic pyeloplasty was discouraged for patients younger than 6 months. Kutikov et al. subsequently published their outcomes of laparoscopic pyeloplasties in children young than 6 months of age, using 3 mm instruments, showing good results and challenging the conclusion that infants are not candidates for minimally invasive approaches (55). Several other series corroborated the feasibility, safety and good outcomes of laparoscopic management for UPJO in infants (56–61). However, all authors acknowledged the technical difficulty of such procedure and advocate for the need of an experienced surgeon and team in order to perform this surgery.

While conventional laparoscopic pyeloplasty has not gained popularity due to its longer learning curve and high technical demands (62), robotic surgery has become vastly more popular due to instruments that allow for 7 degrees of motion, thee-dimensional displays with magnification, tremor reduction and surgeon ergonomics (63). Furthermore, the learning curve for robotic pyeloplasty has been shown to be similar to that of open surgery (64).

In a study comparing robotic to laparoscopic pyeloplasty in children of all ages, Lee et al. were the first to report on the feasibility of the robotic approach in infants (52). The first published series of robotic pyeloplasties just in infants, Kutikov et al. reported resolution of hydronephrosis in seven of nine, while the remaining two patients had no evidence of obstruction on diuretic renography (65). Dangle and coworkers (66) were the first to compare 10 infants who underwent open pyeloplasty to 10 who underwent robotic surgery. They showed similar outcomes with improved aesthetic results and pain control. These authors also recommended using 8-mm instruments instead of the 5-mm ones, which due to their goose-neck joint design require a greater distance from the tissues to fully articulate, reducing the functional space. However, in the hands of an experienced surgeon, 5 mm instruments do not increase either total operative time nor console time when used in infants, compared to larger children (67). The largest-to-date series on robotic pyeloplasty in infants is a multi-center report that includes 62 surgeries in 60 patients, with a mean age of 7.3 months and a median weight of 8.1 kg (68). Resolution or improvement in hydronephrosis was documented in 91% of kidneys and only two patients required redo pyeloplasty, with no intra-operative and only 7 (11%) post-operative complications.

The robotic approach is however under critique for the perceived increase in medical costs, especially when compared to open surgery. Data show that even if robotic equipment increases costs, the shortened post-operative stay and the frequent usage of the system eventually lead to savings (69). In addition, a shorter hospital stay translates into an increased human capital gain for the parents (70). Further data have that robotic surgery is not more expensive than pure laparoscopic pyeloplasty (71). Robotic costs will continue to decrease as more surgeries are performed with this approach. Indeed, between 2003 and 2015, the utilization of robotic pyeloplasty increased by 29% annually. However, this growth was mainly in children and adolescents. While 40% of pyeloplasties in children and adolescents in 2015 were performed robotically, 85% of infant cases were still performed via an open approach (72). Infant patients accounted for 2% and 19% of all robotic and laparoscopic pyeloplasties performed (73), but these trends are likely to change as more surgeons are trained in minimally invasive approaches.

In the end, surgical experience and volume with either open or minimally invasive technique should be a significant factor in the approach chosen to treat UPJO. In the largest report of minimally-invasive pyeloplasties, including 575 pure laparoscopic and robotic cases, a prolonged operative time, but not patient age, was associated with higher complication rates (74). However, success rates were similar. While operative time can be increased by surgical difficulty, it is also a proxy of surgical experience, since progression on the learning curve is associated with shorter operative times (75). Nationwide data from 2008 to 2010 stressed the importance of hospital volume with regards to outcomes of pyeloplasties (76). At high volume centers, peri-operative outcomes of open or minimally-invasive pyeloplasties were similar, however children who underwent minimally-invasive surgery had a shorter hospital stay. Furthermore, the worse outcomes were seen in patients undergoing minimally invasive surgery at low volume centers. Luckily, the same data showed that minimally-invasive pyeloplasties in infants are more frequently performed in high-volume centers than low-volume ones (2.8 vs. 0.4%).

## Technical Considerations for the Robotic Approach in the Infant Patient

**Figure 5:**
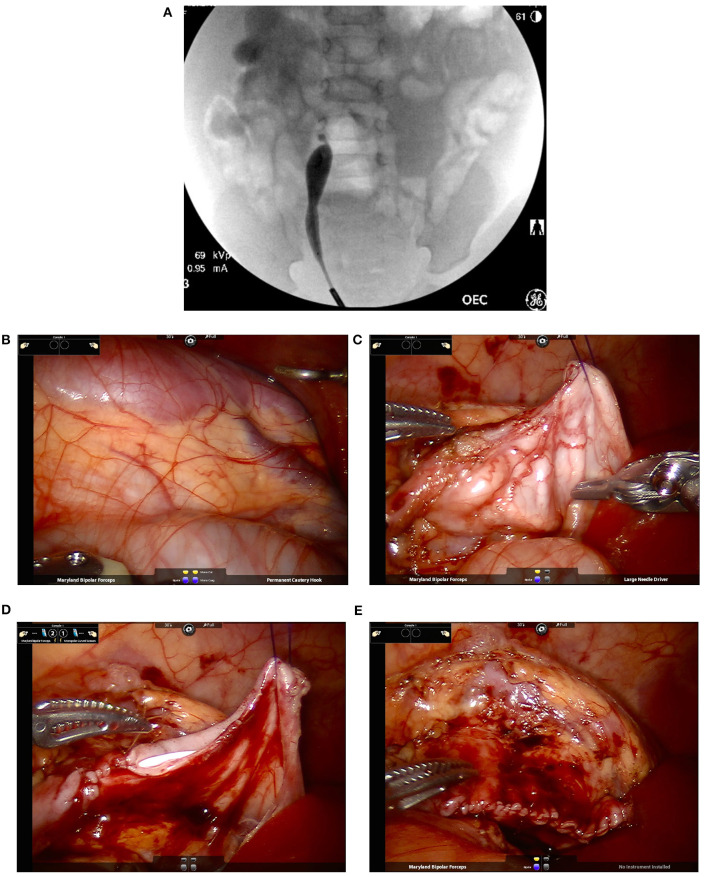
Intra-operative pictures and findings of robotic-assisted laparoscopic pyeloplasty in an infant. **(A)** A retrograde pyelogram performed at the beginning of the case allows for localization of the stricture as well as assessment of its length. This allows for easier planning of reconstructive technique (e.g., dismembered pyeloplasty vs. the need of a flap for longer narrow segments). In addition, it allows for detection of possible distal strictures. A stent is placed at the end prior to the robotic portion of the case. **(B)** Identification of the right kidney with a dilated renal pelvis visible underneath the peritoneum. **(C)** Once the renal pelvis and proximal ureter are dissection, placement of trans-abdominal hitch stich facilitate exposure of the operative field while avoiding the potential use of excessive retraction force by robotic instruments. **(D)** Suturing over the ureteral stent that was placed in a retrograde fashion at time of retrograde pyelogram. **(E)** Complete repair. In the case used for the images, a fair amount of redundant renal pelvis was excised.

At the beginning of the procedure, the authors recommend performing a cystoscopy with retrograde pyelogram. This can rule out any potential distal obstruction as well as delineating the exact extension of the UPJ obstruction. This also allows for placement of a stent in a retrograde manner with an extraction string, eliminating the need for a second cystoscopy (Figure 5).

To facilitate port placement in infants we recommend decompression of the bladder with a catheter and the stomach with an orogastric tube as well as a rectal tube to help with colonic decompression. Placement of the ports in the midline can facilitate reduction of instrument clashing. This port configuration can be performed easily with either the daVinci Si or Xi systems. The surgeon should must be aware of the extension of intra-abdominal movements which are amplified externally with the robotic arms. The depth of the ports can also be reduced, placing the proximal thin line on port at the skin level. We strongly recommend using a box-stitch secured to the fascia to facilitate port placement and prevent port slippage. This will also help with port closure at the end of the procedure. The use of a hitch stitch to elevate the renal pelvis can facilitate anastomotic suturing. Finally, since there will be less gas to dissolve the heat from the electrocautery, it is better to reduce the settings to 15 or less. We have found that use of AirSeal® insufflation reduces the problems of fogging significantly.

## Outcomes

Outcomes of minimally invasive pyeloplasty in the hands of experienced surgeons have been excellent. Reported success rates for laparoscopic surgery are 92–99% while for the robotic approach are 94–100%. The range of complications is wider, 6–30% for laparoscopic and 6–33% for robotic, depending on criterion for consideration as a surgical complication (63, 66, 68, 72, 76).

## Conclusions

Prenatal detection of hydronephrosis secondary to UPJO has increased the numbers of asymptomatic cases from which the clinician must discern who will benefit from surgery and who is best observed. The majority of these children will have resolution of their hydronephrosis, however a non-trivial minority will not improve and may be best managed with surgical intervention to preserve renal functional potential. Early signs of worsening obstruction should be caught promptly in order to offer surgical correction, which can be performed safely with a minimally invasive approach in the hands of an experienced surgeon. Leaving significant obstruction untreated for a prolonged period of time can lead to long-term consequences that will manifest later in the life of the child.

## Author Contributions

CP and NP were involved in literature review. NP drafted the manuscript. CP provided critical review of the manuscript.

## Conflict of Interest

The authors declare that the research was conducted in the absence of any commercial or financial relationships that could be construed as a potential conflict of interest.
